# Transcriptome Analysis of Interspecific Hybrid between *Brassica napus* and *B. rapa* Reveals Heterosis for Oil Rape Improvement

**DOI:** 10.1155/2015/230985

**Published:** 2015-09-10

**Authors:** Jinfang Zhang, Guangrong Li, Haojie Li, Xiaobin Pu, Jun Jiang, Liang Chai, Benchuan Zheng, Cheng Cui, Zujun Yang, Yongqing Zhu, Liangcai Jiang

**Affiliations:** ^1^Crop Science Institute, Sichuan Academy of Agricultural Sciences, Chengdu, Sichuan 6110066, China; ^2^School of Life Science and Technology, University of Electronic Science and Technology of China, Chengdu, Sichuan 610054, China; ^3^Institute of Agro-Products Processing Science and Technology, Sichuan Academy of Agricultural Sciences, Chengdu, Sichuan 6110066, China

## Abstract

The hybrid between *Brassica napus* and *B. rapa* displays obvious heterosis in both growth performance and stress tolerances. A comparative transcriptome analysis for *B. napus* (A^n^A^n^CC genome), *B. rapa* (A^r^A^r^ genome), and its hybrid F1 (A^n^A^r^C genome) was carried out to reveal the possible molecular mechanisms of heterosis at the gene expression level. A total of 40,320 nonredundant unigenes were identified using *B. rapa* (AA genome) and *B. oleracea* (CC genome) as reference genomes. A total of 6,816 differentially expressed genes (DEGs) were mapped in the A and C genomes with 4,946 DEGs displayed nonadditively by comparing the gene expression patterns among the three samples. The coexistence of nonadditive DEGs including high-parent dominance, low-parent dominance, overdominance, and underdominance was observed in the gene action modes of F1 hybrid, which were potentially related to the heterosis. The coexistence of multiple gene actions in the hybrid was observed and provided a list of candidate genes and pathways for heterosis. The expression bias of transposable element-associated genes was also observed in the hybrid compared to their parents. The present study could be helpful for the better understanding of the determination and regulation of mechanisms of heterosis to aid *Brassica* improvement.

## 1. Introduction

*Brassica napus *(rapeseed, AACC, 2*n* = 38) is one of the main oil crops used for human consumption and is widely grown in China, Canada, Europe, and Australia and increasingly grown in South America [1].* B. napus* is likely derived from the natural hybridization and genome doubling from its two diploid parents,* B. rapa* (genome AA, 2*n* = 20) and* B. oleracea* (genome CC, 2*n* = 18) along the Mediterranean coastline in Southern Europe approximately 10,000 years ago [2]. However, there is evidence that the genetic diversity in rapeseed has been continuously reduced by extensive breeding efforts [3]. Approaches to increase the genetic variation of rapeseed have been made by introducing the genetic components from related species as* B. napus* can easily cross with one of its ancestral parents,* B. rapa, *to produce viable interspecies hybrids [4]. Due to the high crossability between* B. napus* and* B. rapa*, and low aneuploidy of their interspecific hybrids, different* B. rapa *accessions have been widely hybridized to rapeseed breeding program and novel agronomic traits from* B. rapa* have been successfully transmitted into commercial* B. napus* varieties [5]. Strong heteroses affecting both biomass and seed yield have been observed in hybrids derived from these interspecific crosses [6]. However, the genetic and molecular mechanism of heterosis in the interspecific hybrids has not been investigated.

Heterosis is a prevalent phenomenon in evolution and breeding process of plants [7]. The hybrid offspring obtains the advantages in many agricultural and developmental traits including biomass yield, plant height, vigor, and stress tolerances from their parents, thereby improving the adaptation of the crop and subsequently increasing the global area sown [8]. Although heterosis has been widely utilized, the genetic and molecular basis of heterotic improvements remains unclear. Various genetic models have been proposed for explaining heterosis and include dominance, overdominance, and pseudo overdominance [9]. Heterotic interactions in the tetraploid* B. napus *can be derived from two types of allelic interactions, namely, those between the genomes of the two diploid progenitors as well as those between the two parental genomes making up a hybrid. Gene expression is potentially altered not only due to a dosage effect but also with gene functional divergence [8]. Epigenetic modification in hybrids genomes can also account for heterosis [10]. Emerging new technologies make it possible to investigate the DNA sequence, RNA transcripts, proteins, and metabolism products genome-wide, allowing for new insights into benefits of heterotic recombinations [7, 9]. Next generation sequencing technology utilizing RNA-seq has emerged as a powerful tool for investigating gene expression data at the whole genome level. Both the gene transcript and expression levels can be detected in RNA-seq which could provide new insights into the molecular mechanisms of heterosis [11].

Chinese cabbage,* Brassica rapa* (syn.* B. campestris*), contains two subspecies named* pekinensis* and* chinensis. *These two distinct groups are used as leafy vegetables widely across eastern Asia and have much genetic variation. In particular,* B. rapa* ssp.* chinensis *has wide morphological variation and is found in China, Korea, and Japan [12]. Recently, a number of genes responsible for developmental and stress tolerances were isolated [13–15], showing that* B. rapa *ssp.* chinensis *could be a novel resource for* B. napus* improvement.

In this study, the transcriptomes of* B. rapa* ssp.* chinensis* Makino,* B. napus,* and their interspecific hybrid were sequenced to investigate the molecular basis of heterosis. Gene expression profiles were used to identify the number of differentially expressed genes, and gene expression levels were analyzed and compared among two parents and their hybrid. This data was used to give a better understanding of additive and nonadditive effects of genes and how these contribute to heterosis, thus providing new insights into the genetic and epigenetic mechanism of subgenomic heterosis in* B. napus*.

## 2. Materials and Methods

### 2.1. Sample Preparation and RNA Isolation

Three lines were grown in a green house at 22°C under 12 hours of light and 12 hours of darkness. Leaves of the seedling were harvested after 4 weeks by cutting off the sixth leaf of each seedling. The leaves of four plants were combined for RNA extraction. Samples were immediately frozen in liquid nitrogen and stored at −80°C until RNA was extracted. Total RNA of each sample was isolated using RNAprep Pure Plant Kit (Tiangen, Shanghai, China). RNA quality was characterized initially by NanoDrop ND1000 spectrophotometer (NanoDrop Technologies, Wilmington, DE, USA) and then further assessed by RIN (RNA Integrity Number) value (>9.5) using Agilent 2100 Bioanalyzer (Santa Clara, CA, USA). Equal quantities of high-quality RNA from each sample were pooled for cDNA synthesis. Cytological observation of the F1 hybrid was performed as described by Zhan et al. [16].

### 2.2. cDNA Library Construction for Illumina Sequencing

The cDNA library was constructed following the manufacturer's instructions of mRNA-Seq Sample Preparation Kit (cat. number RS-930-1001, Illumina, Inc., San Diego, CA). Briefly, the poly(A) mRNA was isolated from total RNA samples with Magnetic Oligo (dT) Beads. The mRNA was then fragmented into small pieces using RNA fragmentation kit (Ambion). Using these short fragments as the templates, the first cDNA strand was synthesized using random hexamer primers and reverse transcriptase (Invitrogen), and the second-strand cDNA was synthesized using DNA polymerase I and RNase H. The cDNA fragments were purified using the QiaQuick PCR extraction kit (Qiagen) and resolved with EB buffer for end reparation and poly(A) addition. The short fragments were then connected with sequencing adapters, and the products were subsequently purified and amplified via PCR. Libraries were prepared from a 400–500 bp size-selected fraction following adapter ligation and agarose gel separation. The quality control analysis on the sample library was performed to quantify the DNA concentration and validate the library. After validation with an Eppendorf Mastercycler Real-Time PCR System, the cDNA libraries were sequenced on the Illumina Hiseq 2000 platform with read length of 2 × 100 bp. The sequencing-derived raw image data were transformed by base calling into sequence data using Illumina Pipeline Software v1.6. The raw sequencing reads have been submitted to NCBI Short Read Archive under the accession number of SRR2131203, SRR2134440, and SRR2134444.

### 2.3. Sequence Data Analysis and Expression Analysis

The raw reads were cleaned by removing adapter sequences, low-quality sequences (reads with ambiguous bases “*N*”), and reads with more than 10%  *Q* < 20 bases. To analyze transcript abundance levels, the uniquely mapped reads for a specific gene were counted by mapping reads to* de novo* assembled distinct sequences using SOAP2 software [17], and the RPKM (Reads Per Kb per Million reads) values were computed as proposed by Mortazavi et al. [18]. Gene transcript abundance differences were obtained from RPKM values using a method modified from Audic's proposal [19]. Gene expression levels were calculated with the RPKM method which is able to eliminate the influence of different gene lengths and sequencing discrepancy within the calculation of gene expression.

### 2.4. Statistics

Statistical analysis of the phenotypic data was performed with a Welch two-sample *t*-test in R (R Development Core Team 2005) [20]. Midparent heterosis (MPH) values were calculated using the formula MPH = (h − (P1 + P2)/2)/((P1 + P2)/2)*∗*100 + 100 (whereas h is the value of the hybrid and P1 and P2 are the values of the one and the other parental line). To test for significance of MPH values the contrast h − (P1 + P2)/2 was used [21].

### 2.5. Quantitative RT-PCR Validation of Differentiation Gene Expression

RNA-seq data were further validated using quantitative real-time PCR analysis (qRT-PCR) for a selected number of genes using gene-specific primer sets. The plant leaves and growth condition for total RNA extraction used for qRT-PCR were the same as these for RNA-seq experiments. Three independent RNA extractions per sample were performed for biological replicates. The reverse transcribed into cDNA using PrimeScritH RT reagent kit with gDNA Eraser (Takara, Dalian, China). Primer pairs of 10 unigenes were designed using Invitrogen's (Carlsbad, CA, USA) OligoPerfect Designer software [22]. Specificity of the primer sets and their product length was verified by agarose gel electrophoresis. The qRT-PCR reaction mixture consisted of the SYBR Premix EX-Taq II Kit (TakaRa, Japan) on iCycler iQ (BIO-RAD) for three repeats of each sample. The thermal cycling conditions were as follows: 95 uC 2 min and 40 cycles at 95°C for 10 s for denaturation and 65°C 20 s and 72°C 30 s for annealing and extension. The expression of* TIPS-41* with primer sequences 5′-TGAAGAGCAGATTGATTTGGCT-3′ and 5′-ACACTCCATTGTC AGCCAGTT-3′ was used as an internal control for normalization to compare the gene expression level between the accessions. The relative levels of gene expression were calculated using the 2^−ΔΔCt^ method.

## 3. Results

### 3.1. Sample Preparation, Sequencing, and Data Filtering

**Figure 1:**
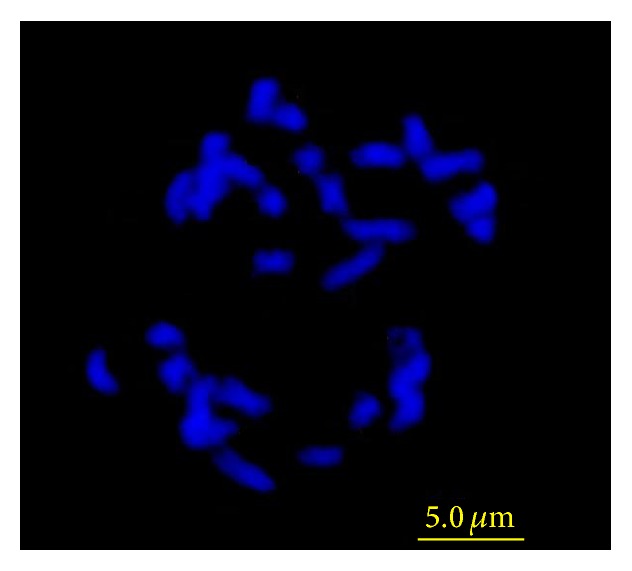
Root tip squash showing chromosomes from the F1 hybrid (chromosome number of 29).

**Figure 2:**
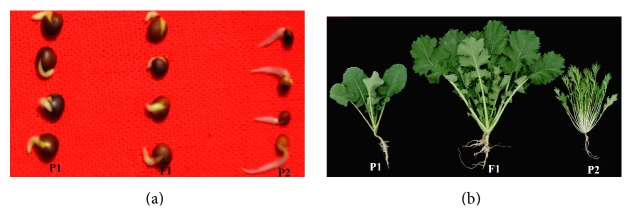
Seed germination (a) and 2-week-old seedlings (b) of P1, P2, and F1 germplasm.

The hybridization of* B. napus* cv. CWH-2 (P1) and* B. rapa* ssp.* chinensis* cv. Qianjin (P2) was made in 2010-2011 oilseed growing season. Root-tip chromosome assessments of F1 showed that all F1 plants had a chromosome number of 29 (Figure 1), suggesting that they were hybrids. The seeds' sizes of F1 hybrids were intermediate to those of the parents, but the seed color resembled that of* B. napus *(Figure 2(a)). Root growth of the F1 over the first 24 h was similar to P1. Leaf morphology of the F1 was more similar to* B. rapa* than to* B. napus* (Figure 2). In 4-week-old seedlings, the F1 plant displayed stronger roots than either of the parents (Figure 2(b)) while adult F1 plants displayed the similar heading time to* B. napus*.

**Figure 3:**
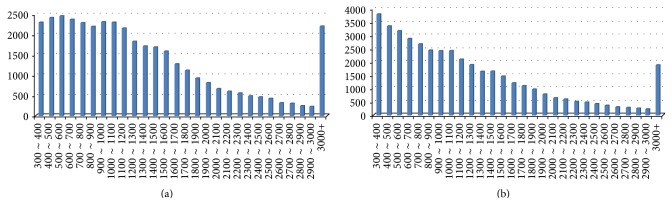
The size distribution of transcriptomic unigenes assembled by the* B. rapa *(a) and* B. oleracea* (b).

To identify transcripts of P1, P2, and F1, three cDNA libraries were constructed from their leaves and sequenced by Illumina paired-end sequencing. Sequence data from each of the libraries consisted of 6.0 Gb from* B. rapa* (AA genome), 5.2 Gb from* B. napus *(AACC genome), and 6.7 Gb from the hybrid (AAC genome). High-quality cleaned raw reads from all of those data were aligned to reference sequences. In total, 67,951,208 reads and 46,207,184 reads were mapped in the* B. rapa *and* B. oleracea* reference genomes, respectively. In the* B. rapa *genome, 38,522 unigenes were assembled with a minimum scaffold size of 200 bp and a total length of 51,995,312 bp, with an average length of 1,349 bp (Figure 3(a)). The* B. oleracea* genome had a total of 42,009 unigenes with a minimum scaffold size of 300 bp, a total length of 51,691,329 bp, and an average length of 1,230 bp (Figure 3(b)).

### 3.2. Heterosis of Differential Gene Expression in* B. rapa*,* B. napus*, and Their Hybrid

The numbers of expressed gene in each genome varied slightly. There were 29,579 (71.8%), 27,939 (67.9%), and 29,447 (71.7%) from* B. rapa, B. napus,* and the hybrid genome, respectively. The unique mapping rates were similar among three* Brassica* samples, suggesting that their genomes were quite alike. Some genes had expression that was germplasm specific. The* B. rapa* genome had approximately about 6% (1,639) of genes that were unique, whilst* B. napus* and the hybrid had only 2% (420) and 3% (951) unique sequences, respectively. There were 25,985 genes expressed in all three lines. A total of 489 genes were expressed in both* B. rapa* and* B. napus* but silenced in the hybrid. Gene expression profiles of the three lines revealed that the number of expressed genes did not show obvious difference in the three sets of material. The mode of gene action for the differentially expressed genes (DEGs) was analyzed. About 27.00% genes (1840 of 6816) exhibited an expression pattern that was not distinguishable from additivity, while the other 72.56% (4946 of 6816) of genes showed nonadditive expression patterns (Table 1). The nonadditive number of DEGs from the cross was further classified into five distinct classes: high-parent dominance (HPD), low-parent dominance (LPD), overdominance (ODO), underdominance (UDO), and partial-dominance. There were 7% of transcripts (545) that were absent in their parents, indicating that there was transcriptional activation of new genes in hybrid F1. There was also transcriptional silencing with 3.2% (218) of parentally expressed gene not being expressed in the hybrid.

**Figure 4:**
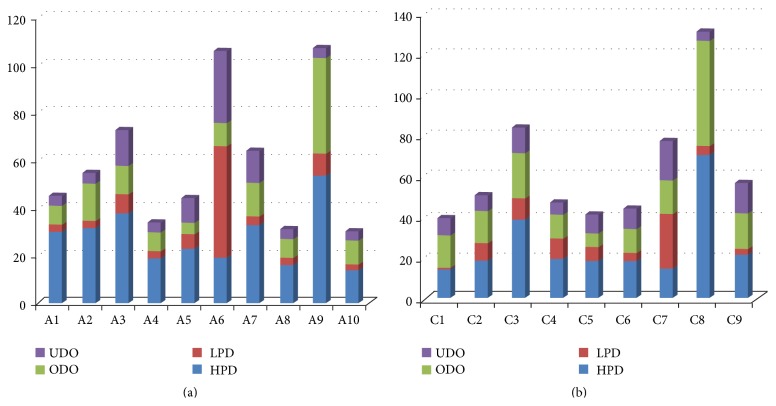
Chromosomal distribution of nonadditive differentially expressed genes with high-parent dominance (HPD), low-parent dominance (LPD), overdominance (ODO), and underdominance (UDO) on the A genome (a) and C genome (b).

There were 2894 unigenes that mapped to the A genome, 2107 that mapped to the C genome (Figure 4), and 593 that were not assigned to either genome. Chromosomal distribution of DEG numbers in hybrids showed that low-parent domiance (LPD) genes were significantly accumulated on chromosomes A06 and C07 (*P* < 0.001), while high-parent dominance (HPD) genes were on chromosomes A09 and C08 (*P* < 0.01). Gene expression variation caused by interspecific hybridization was not randomly distributed along the chromosomes. Therefore, different* B. napus*-*B. rapa* chromosome introgression lines can be developed to the further study of heterosis pertaining to specific chromosomes.

### 3.3. Contribution of A and C Subgenomes to Gene Differential Expression in the Hybrid

**Figure 5:**
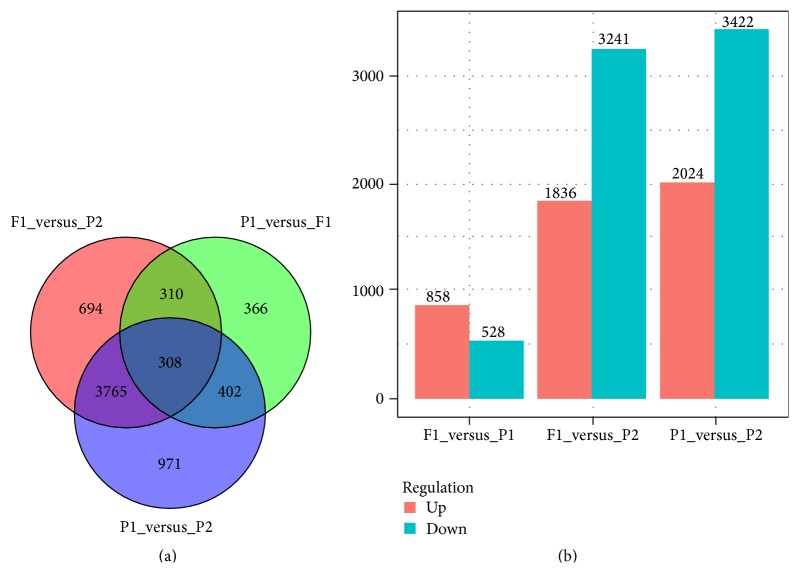
Total numbers of differentially expressed genes between P1, P2, and F1 by Venn diagram analyses (a) and statistics of up- or downregulated genes (b).

**Figure 6:**
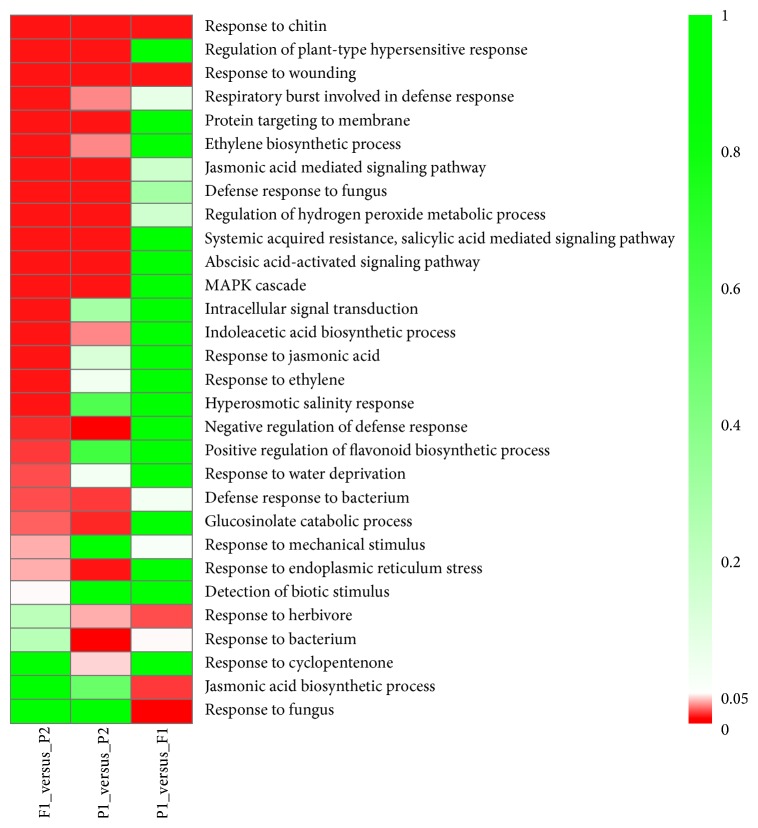
The heat map from GO enrichment analysis of DEGs between the P1, P2, and F1 combinations. Color scale represents *P* values of enrichment test.

There were 1386 significant differentially expressed genes between P1 and F1 samples, 858 genes had increased expression in the F1, and 528 genes had decreased expression. There were 5077 significant differentially expressed genes between P2 and F1 samples, with 3241 genes having increased expression in the F1 and 1836 genes being decreased in expression (Figure 5). Changes to expression from P2 were approximately three times the number of genes compared to from P1. There were more genes with increased expression in the F1 (4098) than with decreased expression (2464) when compared to expression levels in the respective parents. We performed gene ontology (GO) enrichment analysis to test the functional categories for DEGs among P1, P2, and F1 (Figure 6) by AgriGO online tool [23] and R statistical software [20]. There were 10, 12, and 18 functional categories of these transcripts that belonged to the cell component (CC), molecular function (MF), and biological process (BP), respectively. The GO analysis showed that the composition of functional pathways associated with genes showing differential expression was similar when comparing changes from either P1 or P2 with F1.

The biological process category was further subdivided into functional classes in those DEGs with high-parent dominance (HPD, *P* < 0.5) based on gene ontology.  Table 2 highlights the 18 gene ontology classes that were differentially expressed in the F1. Combined with the results in Figure 6, we found that the GOs such as regulation of transcription, metabolic process, defense response, multicellular organismal development, and transporters were enriched in A genomes transcripts. The genes responsible for stress were striking enriched in the differential expressed genes from C genome. It possibly implied that the hybrid expressed protein-coding genes from A genomes of both* B. rapa and B. napus* potentially participated in metabolism and development, while those from C genome of* B. napus* were largely involved in stress resistance.

### 3.4. Transposon Active or Inactive in Hybrid

Compared to the* B. rapa* genome, there were 25 transposable elements (TE) like genes expressed in P1 and P2, while the 11 (44%) genes displayed differential expression in F1 (Table 3). The En/Spm type of transposon, and the retrotransposon-like transcript were significantly enriched. It is likely that the interspecific hybrids significantly modify the transcription of transposon-like genes, which may directly or indirectly influence gene expression of other genes in the heterotic material.

### 3.5. Validation of Transcriptome Data by qRT-PCR

**Figure 7:**
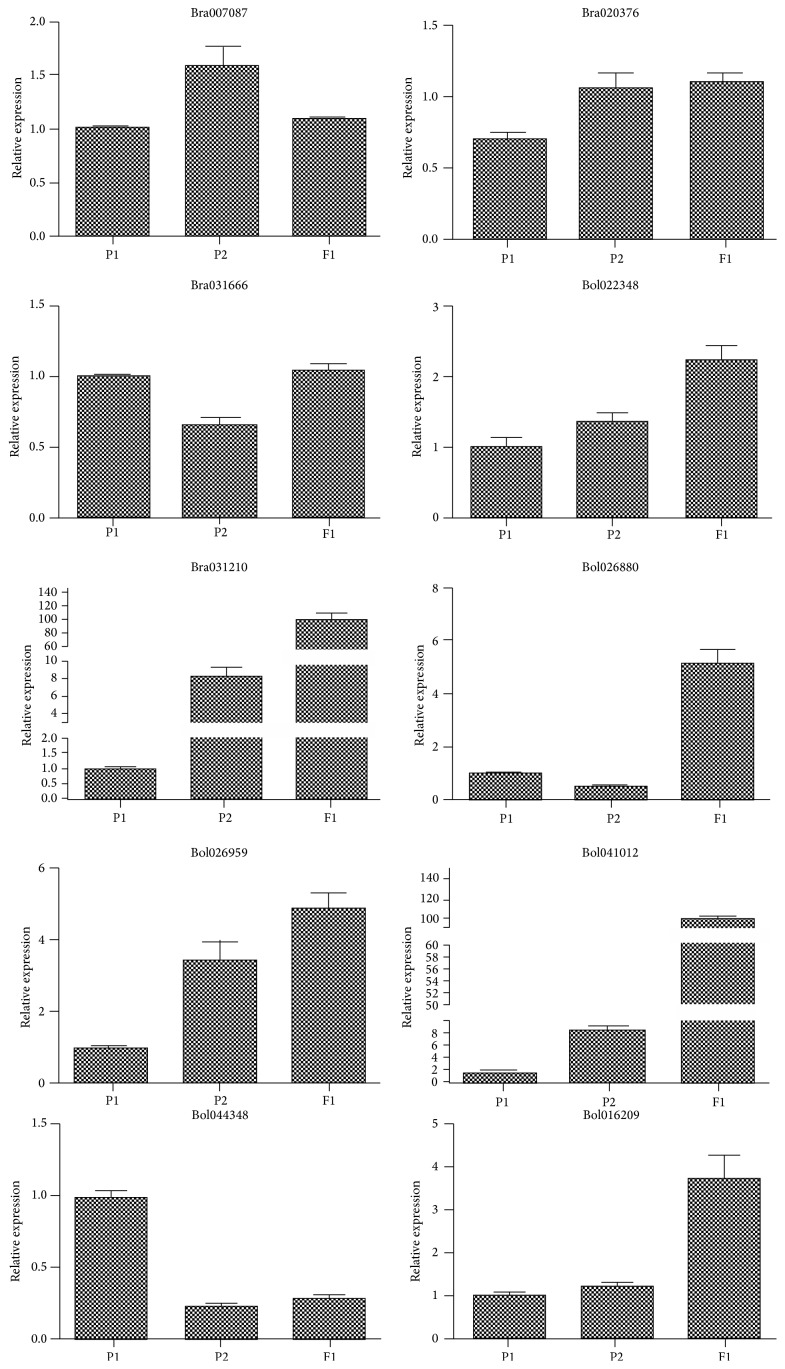
Genes with expression levels validated by qRT-PCR.

To assess the accuracy of RNA-seq data, ten differentially expressed unigenes including stress responsive genes, secondary metabolism biosynthesis genes, and epigenetic modifying genes were selected. Three genes belong to underdominance (UDO) and seven genes are belonging to overdominance (ODO) types, which are represented for heterosis analysis. Primer pairs of 10 unigenes for qRT-PCR were designed and listed in Table 4. We tested the similarity between differential gene expression identified by transcriptome and those identified by qRT-PCR. As shown in Figure 7, the qRT-PCR revealed that 8 of 10 genes (except Bol022348 and Bol026880) that showed the differential gene expression level agreed well with the expression patterns of DGE data. Hence, the qRT-PCR results showed general agreement with their transcript abundance changes determined by RNA-seq, which suggested the reliability of the transcriptomic profiling data among the three samples.

## 4. Discussion

Interspecific hybridization is an important approach to obtaining and utilizing novel agronomic traits from related species for crop improvement. Previous researches revealed strong heterosis between* B. napus* and* B. rapa* on biomass and seed yield [5–7]. Recently, Li et al. [24] reported that* B. napus*-like individuals in the F3 and F4 generations, from interspecific hybridization between* B. napus* and* B. oleracea*, showed diverse genetic variation relative to current* B. napus* and strong heterotic potential. We selected a number of backcrossed offspring derived from the F1 by being crossed with P1 (*B. napus*) and new traits such as wrinkle leaves, six sepals which different from their parents were observed in the progenies. In particular, we identified several lines which displayed high yield up to 4,500 kg per hectare in BC1F2 population. It suggested that the novel lines from interspecific hybridization between* B. napus* and* B. rapa* with high yield good quality can be potentially useful for oilseeds production in the near future.

Intraspecific hybrids of* Arabidopsis thaliana *[25, 26], rice [27–29], and maize [30, 31] revealed that there are both additive and nonadditive effects of gene expression in hybrids, and heterosis is strongly influenced by genetic differences between two parents. A transcriptome approach has been utilized to investigate the heterosis of intraspecific hybrids of* A. thaliana*, rice, maize [29, 30]. Our research of the transcriptomes from* B. napus* and* B. rapa* demonstrated the genetic effect of interspecies heterosis at the gene expression level. More genes specific to certain pathways in further experiments are needed to fully validate the heterotic association between the gene expression patterns and the target agronomic traits. In the present study, we found that both additive and dominance effects contributed to interspecies heterosis between* B. napus* and* B. rapa*. Analysis of the number of DEGs in hybrid and parental germplasm revealed that the nonadditive effect contributed more than the additive effect. Previous research reveals interspecies heterosis between* B. napus *and* B. rapa* through genetic analysis but the main focus was on phenotypic variation [6, 32]. Our study provided evidence of differential gene expression and shed new insights on the molecular mechanism for this heterosis in the interspecific hybrids.

*Brassica* species constitute an elite system for investigating the changes in genomic structure and functional divergence of duplicate genes in the process of hybridization and polyploidization among the diploid species and their amphidiploids [32, 33]. Subgenomes in* Brassica* species are designated with superscripts with A^n^ and A^r^ representing A genomes from* B. napus* and* B. rapa*, respectively [33]. We have shown intersubgenomic heterosis between A^r^ and A^n^ genomes, supporting work by others that show that such heterosis can be useful in creating genetic diversity and be used in breeding. Studies indicated that the midparent heterosis contributes intersubgenomic heterosis [8, 34, 35]. Therefore, the utilization of intersubgenomic heterosis can be creating genetic diversity and breeding. In the present study, we found that the nonadditive DEGs from the A genome in the hybrids mainly participated in metabolism and development, while those from the C genome were largely involved in stress resistance. It is likely that the neofunctionalization of subgenome contributes the heterosis or the genomic adaptation in hybrid. Recently, Li et al. [36] investigated the total homoeolog expression level of hexaploid wheat (AABBDD) and compared it to its ancestral parents* T. turgidum* (AABB) and* Ae. tauschii* (DD), finding that genes expressed from the AABB potentially participated in development and those from* Ae. tauschii* (DD) were more likely involved in adaptation. Differential contributions from the parental sources may also be occurring in the* Brassica* hybrids, and this may hint at the molecular mechanisms behind heterosis. Transposable elements (TEs) have major effects on different plant genomes and can significantly contribute differences in genome size of over 1,000-fold [37]. Recent sequencing of the* B. rapa* and* B. oleracea* genomes revealed that the amplification of TEs is one of the main factors inducing the difference in genome size [37]. The overall TEs in either* Brassica *genomes are expressed at very low levels, and the expression levels of different TE categories and families vary among different organs [38]. Recently, the variations in TE-associated sequences during the process of allopolyploidisation were detected between* Brassica rapa* (AA) and* B. oleracea* (CC), as well as in successive generations of self-pollinated progeny [39]. Our study revealed that the expression activities of TE categories or families were clearly modified in the hybrids between the two* Brassica* species, and different types of TEs showed different patterns of variation during the process of hybridization. Therefore, the association of activation and inactivation of TEs and of TE related genes expression in relation to heterotic gene expression needs to be further investigated.

## 5. Conclusions

Development of interspecific hybrids has been widely exploited for the heterosis breeding of* Brassica* crops. The interspecific hybrid between* Brassica napus* and* B. rapa *displayed obvious heterosis. In the present study, the comparative transcriptome analysis for the parents* B. napus* and* B. rapa* and its hybrid F1 was sequenced using the platform of Illumina/Solexa with* de novo* assemblage. The nonredundant unigenes were identified using* B. rapa* (A genome) and* B. oleracea* (C genome) as reference genomes. The nonadditively differentially expressed genes were potentially related to the heterosis. The results indicated that the differentially expressed genes in hybrids from the A genome mainly participated in metabolism and development, while those from the C genome were largely involved in stress resistance. The coexistence of multiple gene actions and transposable element-associated genes regulations in the hybrid were observed. The present study could be helpful for the better understanding of the mechanisms of heterosis, and a list of candidate genes will be used for future* Brassica* breeding.

## Figures and Tables

**Table 1:** Summary of dominance patterns of differentially expressed genes.

DGs	Number	Percentage
Additivity	1840	27
Nonadditivity	4946	72.56
Others	30	0.44
High-parent dominance (HPD)	353	7.14
Low-parent dominance (LPD)	191	3.86
Overdominance (ODO)	593	11.99
Underdominance (UDO)	193	3.9
Positive partial-dominance (PPD)	1635	33.06
Negative partial-dominance (NPD)	1981	40.5
Total	6816	

Additivity: F1 ≈ 1/2(P1 + P2); nonadditivity: F1 > 1/2(P1 + P2) or F1 < 1/2(P1 + P2). High-parent dominance (HPD): F1 ≈ P1 > P2 or F1 ≈ P2 < P1; low-parent dominance (LPD): F1 ≈ P1 < P2 or F1 ≈ P2 < P1; overdominance (ODO): F1 > P1 and F1 > P2; underdominance (UDO): F1 < P1 and F1 < P2.

**Table 2:** Significant gene ontology terms of high-parent dominance (HPD) in the biological process category from A and C genomes.

Gene ID	Gene O terms
Bra028791	Regulation of transcription (GO:0006355)
Bra005111	Metabolic process (GO:0008152)
Bra032185	Defense response (GO:0002679)
Bra010724	Multicellular organismal development (GO:0007275)
Bra007111	Metabolic process (GO:0010408)
Bra031515	Single-organism transport (GO:0044765)
Bra031678	Transport (GO:0006810)
Bra027171	Transmembrane transport (GO:0006855)
Bra024033	Peroxidase activity (GO:0004601)
Bra002550	Chromosome segregation (GO:0007059)
Bol015697	Response to molecule of bacterial origin (GO:0002237)
Bol032712	Defense response to insect (GO:0002213)
Bol014351	Defense response to bacterium (GO:0042742)
Bol045822	Defense response (GO:0002679)
Bol034224	Response to wounding (GO:0009611)
Bol038369	Single-organism process (GO:0044699)
Bol045822	Regulation of transcription (GO:0006355)
Bol015697	Response to molecule of bacterial origin (GO:0002237)

**Table 3:** The differential expressed transposon-like genes in hybrid F_1_.

Gene ID	Regulation	Chromosome	Similar to transposon
B.rapa.newgene.1068	Inactive	A09	Retrotransposon-like protein (*Arabidopsis thaliana*)
B.rapa.newgene.871	Inactive	A07	Putative transposon protein (*Arabidopsis thaliana*)
B.rapa.newgene.1142	Inactive	A09	Retrotransposon-like protein (*Arabidopsis thaliana*)
B.rapa.newgene.957	Inactive	A08	Retrotransposon (*Arabidopsis thaliana*)
Bra040302	Up	A08	Putative transposon protein (*Arabidopsis thaliana*)
B.rapa.newgene.307	Up	A03	En/Spm transposon protein (*Arabidopsis thaliana*)
B.rapa.newgene.726	Up	A06	En/Spm-like transposon protein (*Arabidopsis thaliana*)
Bra034711	Active	A05	hAT transposon superfamily protein (*Arabidopsis thaliana*)
Bra027867	Active	A09	Pol polyprotein transposon element Bs1 (*Zea mays*)
B.rapa.newgene.8	Active	A01	Tam1 transposon protein (*Arabidopsis thaliana*)
Bra035011	Active	A07	Putative transposon protein (*Arabidopsis thaliana*)
Bol019342	Inactive	C09	Retrotransposon Tto1 DNA (*Nicotiana tabacum*)
Bol041429	Up	C07	Putative Tam3-like transposon protein (*Zea mays*)
Bol035479	Down	C03	En/Spm-related transposon protein (*Brassica oleracea*)
Bol005562	Down	C03	En/Spm-related transposon protein (*Brassica oleracea*)
Bol019765	Down	C09	En/Spm-related transposon protein (*Brassica oleracea*)
Bol006373	Down	C07	Transposon protein-like (*Arabidopsis thaliana*)
Bol035958	Up	C02	Transposon-like ORF (*Brassica oleracea*)
Bol030703	Active	C03	Retrotransposon Tto1 DNA (*Nicotiana tabacum*)
Bol030374	Active	C09	En/Spm-related transposon protein (*Brassica oleracea*)
Bol004880	Active	C04	Retrotransposon Tto1 DNA (*Nicotiana tabacum*)
Bol043208	Active	C07	Similar to retrotransposon (*Arabidopsis thaliana*)

**Table 4:** The qPCR primers for selected genes.

Unigenes	Primers	Biological process	Nonadditivity
Bra007087 F	TGCAGCGCTTGATTTACCT	Oxidation-reduction process	HPD
Bra007087 R	GCAAACTCCAGAGCTATGT		
Bra020376 F	AGGTCATTCTGGTGAGCCACA	Chaperones	HPD
Bra020376 R	TGGAGACTTTGGAAGGATACT		
Bra031210 F	ACGAGGCTCAGTCTCGTGGT	Response to water deprivation	ODO
Bra031210 R	TCCGCTGCGGTATCCACCA		
Bra031666 F	TGCCAAGGACAACAACTTGGACT	Lignin biosynthetic process	HPD
Bra031666 R	AGTTGGTTGTAGGACTGGTCCA		
Bol016209 F	TAACCTACCAGAAGCACGGT	Response to abscisic acid	ODO
Bol016209 R	ACAACTTCAACGGTGCACGACT		
Bol022348 F	AGGTACCTTACGAGTCTCGT	Response to karrikin	HPD
Bol022348 R	CACGAACCTGATAGAAGCTCGT		
Bol026880 F	TGTAAGGCTACGAAGGGACAT	Defense response to fungus	HPD
Bol026880 R	TCCAACATATCCATATGTCCGT		
Bol026959 F	TAGCTCTTCCTCTTCAAGCGAT	Defense response to fungus	ODO
Bol026959 R	TCCTTCTTCCTCTTCTCACCA		
Bol041012 F	TGACACATTGTGGATGGAACT	Cellular hyperosmotic response	ODO
Bol041012 R	GGCATACCAATCATTGGAACT		
Bol044348 F	TACTACTGGACCTTTGGTGCT	Calcium ion transport	HPD
Bol044348 R	GATCTTCATCTGAAGGTCACT		
